# Sequential therapy with supercharged NK cells with either chemotherapy drug cisplatin or anti-PD-1 antibody decreases the tumor size and significantly enhances the NK function in Hu-BLT mice

**DOI:** 10.3389/fimmu.2023.1132807

**Published:** 2023-05-01

**Authors:** Kawaljit Kaur, Po-Chun Chen, Meng-Wei Ko, Ao Mei, Emanuela Senjor, Subramaniam Malarkannan, Janko Kos, Anahid Jewett

**Affiliations:** ^1^Division of Oral Biology and Medicine, The Jane and Jerry Weintraub Center for Reconstructive Biotechnology, University of California School of Dentistry, Los Angeles, CA, United States; ^2^Laboratory of Molecular Immunology and Immunotherapy, Blood Research Institute, Versiti, Milwaukee, WI, United States; ^3^Department of Biotechnology, Jožef Stefan Institute, Ljubljana, Slovenia; ^4^Faculty of Pharmacy, University of Ljubljana, Ljubljana, Slovenia; ^5^Department of Microbiology and Immunology, Medical College of Wisconsin, Milwaukee, WI, United States; ^6^The Jonsson Comprehensive Cancer Center, University of California, Los Angeles (UCLA) School of Dentistry and Medicine, Los Angeles, CA, United States

**Keywords:** NK cells, supercharged NK cells, cytotoxicity, IFN-γ, chemotherapeutic, Hu-BLT, check-point inhibitor

## Abstract

**Introduction and methods:**

In this study we report that sequential treatment of supercharged NK (sNK) cells with either chemotherapeutic drugs or check-point inhibitors eliminate both poorly differentiated and well differentiated tumors *in-vivo* in humanized-BLT mice.

**Background and results:**

sNK cells were found to be a unique population of activated NK cells with genetic, proteomic, and functional attributes that are very different from primary untreated or IL-2 treated NK cells. Furthermore, NK-supernatant differentiated or well-differentiated oral or pancreatic tumor cell lines are not susceptible to IL-2 activated primary NK cell-mediated cytotoxicity; however, they are greatly killed by the CDDP and paclitaxel in in-vitro assays. Injection of one dose of sNK cells at 1 million cells per mouse to aggressive CSC-like/poorly differentiated oral tumor bearing mice, followed by an injection of CDDP, inhibited tumor weight and growth, and increased IFN-γ secretion as well as NK cell-mediated cytotoxicity substantially in bone marrow, spleen and peripheral blood derived immune cells. Similarly, the use of check point inhibitor anti-PD-1 antibody increased IFN-γ secretion and NK cell-mediated cytotoxicity, and decreased the tumor burden in-vivo, and tumor growth of resected minimal residual tumors from hu-BLT mice when used sequentially with sNK cells. The addition of anti-PDL1 antibody to poorly differentiated MP2, NK-differentiated MP2 or well-differentiated PL-12 pancreatic tumors had different effects on tumor cells depending on the differentiation status of the tumor cells, since differentiated tumors expressed PD-L1 and were susceptible to NK cell mediated ADCC, whereas poorly differentiated OSCSCs or MP2 did not express PD-L1 and were killed directly by the NK cells.

**Conclusions:**

Therefore, the ability to target combinatorially clones of tumors with NK cells and chemotherapeutic drugs or NK cells with checkpoint inhibitors at different stages of tumor differentiation may be crucial for successful eradication and cure of cancer. Furthermore, the success of check point inhibitor PD-L1 may relate to the levels of expression on tumor cells.

## Introduction

Cancer is the second leading cause of mortality globally (1, 2). Because of limited efficacy, and undesirable toxicities of current cancer therapies, there is an urgent need to improve the clinical outcomes in cancer patients (3–5). Despite intense research and improvement in therapeutic regimens, diagnosis of many cancers at the later stages of the disease remains associated with poor prognosis (2). With the rapid advances in the immunotherapy approaches in cancer, there is now greater focus on development of cell-based immunotherapies. More recently, clinical trials on cancer immunotherapies have demonstrated that immunotherapy is an effective treatment modality for many types of malignancies including metastatic melanoma, lung cancer, and bladder cancer (6–10). Effectors of the immune system are thought to shape the survival and maturation of tumor cells and also in the elimination of cancer. Hence, while surgery in combination with chemotherapy and radiotherapy is considered a fundamental therapeutic strategy and the standard of care in many solid tumors, immunotherapy alone or in combination with other therapies is now playing an important role in the treatment of various malignancies. The ultimate goal of immunotherapies is to assist the immune system to eradicate the cancer cells, and it appears that immunotherapy is on the way to transform terminal cancer to perhaps a more manageable chronic disease, and ultimately cure the patients from the disease if underlying mechanisms of immune activation and function are clearly delineated and the role of each immune subset clarified in the shaping of the tumors (11).

Heterogeneity in tumor cells necessitates treatment strategies which target all the different clones of tumor cells, and restores the function of immune cells in patients to prevent recurrences and the generation of new cancers. Thus, combinatorial treatments with immunotherapy may be required to target tumor cells at different stages of differentiation. We have shown in many previous publications that natural killer cells (NK) cells target cancer stem cells (CSCs)/poorly differentiated tumors whereas well differentiated tumors are not susceptible to primary NK cell effects, but they are susceptible to CD8+ T cell function, chemotherapy, radiation and antibody therapy (12). Not too many treatment strategies other than NK cells are capable of targeting CSCs, or poorly differentiated tumors, primarily due to their lack of or much lower expression of MHC-class I (13). We have shown recently that cannabinoids are potentially other factors that can target the CSCs/poorly differentiated oral and pancreatic tumors (14). However, radiation (15–18) and chemotherapy (12, 16–19) were unsuccessful in targeting CSCs. We have also shown that NK cell-mediated ADCC was significantly higher against PD-L1 and MICA/B expressing differentiated tumors as compared to their CSCs (20). It is conceivable that CAR-T and CAR-NK cells generated to high expressing surface receptors on CSCs/poorly differentiated tumors can achieve similar outcomes as NK cells; however, down-modulation or loss of those receptors on these cells may make these CARs ineffective and promote tumor growth and expansion, whereas the more a cell mutates and loses surface receptors, the better it is targeted by the NK cells (21). Indeed, NK cell-based clinical trials have demonstrated not only the safety but also the efficacy in decrease in tumor relapse rate (22–25).

NK cells mediate direct cytotoxicity as well as antibody-dependent cellular cytotoxicity (ADCC) (26). Two effector functions of NK cells that are crucial for the elimination of the tumors are NK cell-mediated cytotoxicity and secretion of cytokines which lead to direct killing of CSCs, and NK cell-mediated differentiation of tumors respectfully (27). IFN-γ and TNF-α secreted by NK cells play a crucial role in differentiating CSCs/undifferentiated tumors (28). We have shown previously that differentiated tumors are favorable targets of chemotherapy, thus, NK cells could assist chemotherapy in eradication of tumors (12, 29). Also, combining NK cell immunotherapy with checkpoint inhibitors such as anti-PD1 have shown promising results (26, 30).

In this study, we demonstrate the differences between the primary and supercharged NK cells (sNK) based on their genetics, proteomics and functional attributes, demonstrating the uniqueness of sNK cells not only for their increased cycling and significant rate of expansion, but also their superior function and their unique transcriptional profile on single cell RNAseq analysis level. The *in-vivo* studies revealed how the combination of sNK cells with chemotherapy or sNK cells with anti-PD1 antibody reduce tumor burden and either restore or increase IFN-γ secretion, and cytotoxic function of NK cells in various tissue compartments of oral and pancreatic tumor-bearing humanized-BLT (hu-BLT) mice. We also provide some underlying mechanisms governing such *in vivo* observations in a series of *in vitro* studies.

## Results

### Unique attributes of supercharged NK cells in comparison to primary NK cells

**Figure 1:**
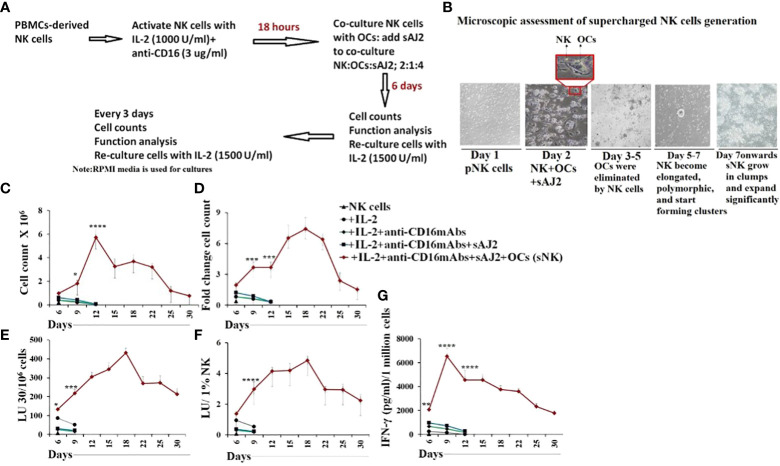
Osteoclasts induced higher cell expansion, increased cytokine secretion and cytotoxicity in NK cells in comparison to IL-2, IL-2+anti-CD16mAbs and IL-2+anti-CD16mAb+sAJ2 treatments Osteoclasts (OCs) were generated as described in the Materials and Methods section. NK cells (0.5x10^6^ cells/2ml) were treated with a combination of IL-2 (1000 U/ml) and anti-CD16mAb (3μg/ml) for 18 hours before they were co-cultured with OCs and treated with sAJ2 (2:1:4: NK : OCs:sAJ2). NK cells were counted on days 6, 9, 12, 15, 18, and onwards until cells are expanding (Average: 24-36 days) **(A, B)**. NK cells (0.5x10^6^ cells/2ml) were left untreated, or treated with IL-2 (1000 U/ml), or a combination of IL-2 (1000 U/ml) and anti-CD16mAb (3μg/ml), or a combination of IL-2 (1000 U/ml), anti-CD16mAb (3μg/ml), and sAJ2 (2:4;NK:sAJ2), or a combination of IL-2 (1000 U/ml), anti-CD16mAb (3μg/ml), sAJ2, and OCs (2:1:4: NK : OCs:sAJ2). NK cells were counted on the days shown in the Figure **(C)**, and the fold change based on the initial cell count of 0.5x10^6^ cells/ 2 mL were determined every 3 days as shown in the figure **(D)**. NK cell-mediated cytotoxicity against oral squamous cell carcinoma stem cell line (OSCSCs) was determined on the days shown in the figure using a standard 4-hour ^51^Cr release assay. The lytic units 30/10^6^ cells were determined using the inverse number of NK cells required to lyse 30% of OSCSCs x 100 **(E)**. Lytic units per 1% NK cells were determined based on the percentages of CD16+/CD56+ NK cells in the cultures obtained by flow cytometric analysis **(F)**. The supernatants were harvested from the cultures on the days shown in the Figure E to determine IFN-γ secretion using single ELISA, and the levels were adjusted based on the number of cells **(G)**. Averages and std. dev of three independent experiments are shown in **Figure 1**. ****(p value<0.0001), ***(*p* value 0.0001-0.001), **(*p* value 0.001-0.01), *(*p* value 0.01-0.05).

**Figure 2:**
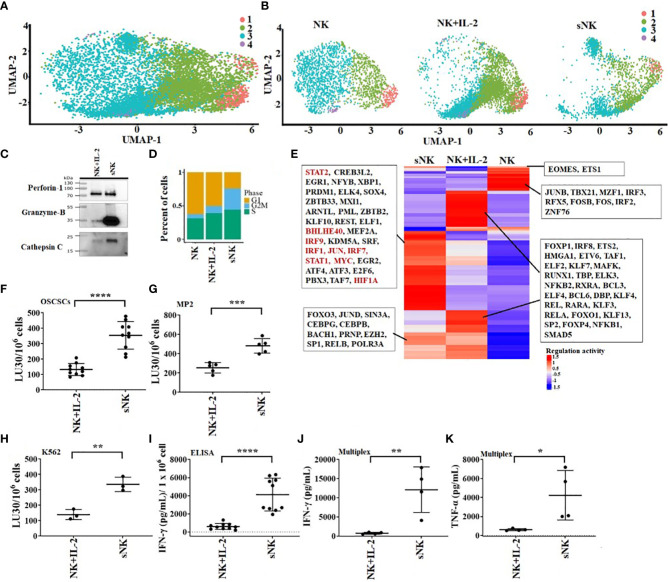
OC-expanded supercharged NK cells exhibit higher levels of cytotoxic granules, increased cytotoxicity and augmented secretion of cytokines when compared to primary activated NK cells. OCs were generated as described in the Materials and Methods section. NK cells (0.5x10^6^ cells/ 2ml) from healthy individuals were treated with a combination of IL-2 (1000 U/ml) and anti-CD16 mAbs (3 µg/ml) overnight before they were cultured with OCs and sAJ2 at a ratio of 2:1:4 (NK:OCs:sAJ2). Untreated NK cells, IL-2 treated NK cells, and super-charged NK (sNK) cells are used to construct single-cell cDNA libraries for sequencing. UMAP of all of the samples combined **(A)** or separated **(B)** are shown. Colors represent different UMAP clusters indicating genetically distinct NK cell subsets. Western blot of protein expression of granzyme B, cathepsin C, and perforin-1 in sNK vs. IL-2 treated primary NK cells derived from the same donor is shown in figure **(C)**. Loading control can be found in **Figure S1A**. Each cell is assigned a cell-cycle score based on gene markers of different phases. The percentage of cells in each phase is represented in the bar-plot for untreated NK cells, IL-2 treated NK cells, and sNK cells **(D)**. SCENIC is used to analyze the regulon activity in each condition. Each row of the heatmap represents a regulon, with some highlighted in the box **(E)**. On day 14 of cultures, another set of NK cells were purified from healthy donors and were treated with IL-2 (1000 U/ml) overnight. Cytotoxicity of day 15 sNK cells and overnight IL-2 treated primary NK cells was determined using standard 4-hour ^51^Cr release assay against OSCSCs **(F)**, MP2 **(G)**, and K562 **(H)**. The Lytic units (LU) 30/10^6^ cells were determined using the inverse number of NK cells required to lyse 30% of OSCSCs (n=10) **(F)** or MP2 (n=5) x 100 **(G)** or K562 (n=3) x 100 **(H)**. Primary NK cells were treated with IL-2 as described in **Figure 1F**, and the supernatants were harvested from day 15 sNK cells or IL-2 treated primary NK cells after an overnight incubation and were used to determine IFN-γ secretion using single ELISA. The amounts of IFN-γ secretion were adjusted based on 1 x 10^6^ cells (n=10) **(I)**. Primary NK cells were treated with IL-2 as described in **Figure 1F**, and the supernatants were harvested from day 15 expanded sNK cells or IL-2 treated primary NK cells after an overnight incubation, and they were used to determine IFN-γ **(J)** and TNF-α **(K)** using multiplex cytokine arrays (n=4). ****(p value<0.0001), ***(*p* value 0.0001-0.001), **(*p* value 0.001-0.01), *(*p* value 0.01-0.05).

In our previous studies as well in this study, we demonstrate the superior ability of osteoclasts (OCs) to condition NK cells for greater expansion and heightened function (**Figure S1**) (31). Here, we compared cell expansion, IFN-γ secretion, and NK cell-mediated cytotoxicity of untreated, IL-2 treated NK, IL-2+anti-CD16mAbs treated NK, and IL-2+anti-CD16mAbs+sAJ2 treated NK cells with IL-2+anti-CD16mAbs+sAJ2+OCs treated NK cells, and found higher cell expansion and increased function in the presence of OCs (**Figure 1**). Probiotic bacteria, sAJ2 is a combination of 7-8 different strains, and is prepared as described previously (32). Due to their unusually high expansion rate and potent function, we coined IL-2+anti-CD16mAbs+sAJ2+OCs treated NK cells as supercharged NK (sNK) cells to differentiate them from all the other NK cell subsets that we had tested in our laboratory throughout the last 30 years (28). To further understand the differences between IL-2 treated primary NK (NK+IL-2) cells and sNK cells, we performed single-cell RNA sequencing. In the analysis of NK+IL-2 and sNK cells, we also integrated untreated NK cells derived from donor PBMC to help characterize the NK cell subsets. By studying untreated NK, NK+IL-2, and sNK cells, we were able to identify 4 transcriptionally unique NK cell clusters (**Figure 2A**). All 4 clusters have a consistent expression of NK cell genetic markers (*IL2RB*, *CD7*, *NKG7*). Among the 4 clusters, Cluster 1 has the highest expression of *IL7R* and *NCAM1*, resembling the transcriptional signature of previously characterized CD56^bright^ NK cells (33). Cluster 3 has a comparably higher expression of genes related to cytotoxicity (*GZMB*, *PRF1*) and *FCGR3A*, which are identified as the genetic signature of cytotoxic CD56^dim^ NK cells. The gene expression pattern of cluster 2 follows a subset of transitional NK cells between CD56^bright^ and CD56^dim^ NK cells. When the single-cell clusters are split by the three conditions, cluster 4 is shown to be exclusive in untreated NK cells (**Figure 2B**). We have also analyzed the expression of the main effector molecules in NK cell cytotoxic granules: perforin-1, granzyme B and cathepsin C (cathepsin C is responsible for the activation of granzyme B) (32). A significantly higher expression of granzyme B and cathepsin C in the presence of slightly decreased perforin was seen in sNK cells in comparison to primary IL-2 treated NK cells (**Figures 2C**, **S2A**). Through the cell-cycle score analysis on the single-cell RNA sequencing result, a considerably higher amount of sNK cells is assigned to the G2M phase, indicating a more active proliferation program in the sNK cells compared to untreated and NK+IL-2 cells (**Figure 2D**). Also, by performing SCENIC analysis on the sequencing data, a distinct regulon network is utilized in the sNK cells compared to either untreated or NK+IL-2 cells. Among the predicted regulon activities, sNK cells have upregulated regulon activities associated with NK cell survival (STAT2, IRF9) and effector functions (IRF1, JUN, STAT1, HIF1A) (**Figure 2E**) (27, 34–37). When assessed NK cell-mediated cytotoxicity of IL-2 treated primary NK and sNK cells against oral squamous carcinoma stem cells (OSCSCs), Mia PaCa-2 (MP2), and K562 cell lines, significantly higher cytotoxicity was mediated by sNK cells in comparison to NK+IL-2 cells (**Figures 2F–H**). We also observed higher secretion of IFN-γ and TNF-α by sNK cells in comparison to primary NK cells treated with IL-2 (**Figures 2I–K**, **S2B**). These results exhibited higher anti-cancer activity of sNK cells in comparison to primary activated NK cells.

### Differentiated oral and pancreatic tumors are more susceptible to chemotherapeutic drugs in comparison to their stem-like counterparts

**Figure 3:**
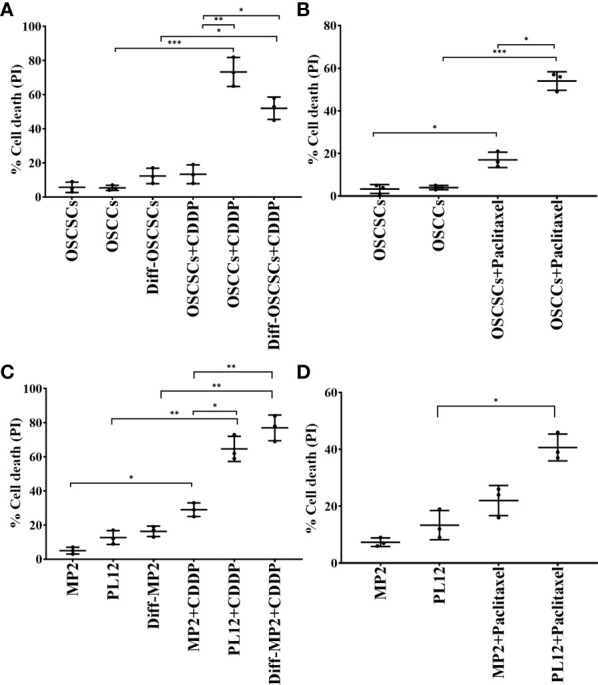
Increased susceptibility of differentiated oral and pancreatic tumor cell lines to chemotherapeutic drugs in comparison to their stem-like counterparts. OSCSCs were differentiated using supernatants from IL-2 (1000 U/ml) and anti-CD16 mAbs (3µg/mL) treated primary NK cells as described in Materials and Methods section. OSCSCs, OSCCs and NK-diff-OSCSCs were treated with cisplatin (60 µg/mL) for 18-20 hours, after which, the cells were stained with propidium iodide (PI) to determine percent cell death using flow cytometric analysis (n=3) **(A)**. OSCSCs and OSCCs were treated with paclitaxel (40 µg/mL) for 18-20 hours, after which, the cells were stained with PI to determine percent cell death using flow cytometric analysis (n=3) **(B)**. MP2 cells were treated with supernatants from IL-2 (1000 U/ml) and anti-CD16 mAbs (3µg/mL) treated primary NK cells in order to induce differentiation as described in the Materials and Methods section. MP2, PL12, and NK-diff-MP2 cells were treated with cisplatin (60 µg/mL) for 18-20 hours, after which, the cells were stained with PI to determine percentage of cell death using flow cytometric analysis (n=3) **(C)**. MP2 and PL12 tumor cells were treated with paclitaxel (40 µg/mL) for 18-20 hours, after which, the cells were stained with PI to determine percent cell death using flow cytometric analysis (n=3) **(D)**. ***(*p* value 0.0001-0.001), **(*p* value 0.001-0.01), *(*p* value 0.01-0.05).

Our previous findings have demonstrated that differentiated tumors were more sensitive to chemotherapeutic drugs in comparison to CSCs/poorly differentiated tumors (12). Here, we determined the extent of cell death of oral cancer stem-like cells (OSCSCs), NK-diff-OSCSCs, CSCs/poorly differentiated pancreatic cancer MP2, and differentiated pancreatic cancer (PL12) and NK-diff-MP2 with or without the treatments with chemotherapeutic drugs cisplatin (CDDP) and paclitaxel (**Figure 3**). We observed higher cell death induced by CDDP (**Figures 3A, C**, **S3**) and paclitaxel (**Figures 3B, D**) against differentiated tumors in comparison to their CSCs/poorly differentiated counterparts. Thus, differentiation of tumors by the NK cells is an important step not only in curtailing the tumor growth, but more importantly in the response to chemotherapy drugs.

### sNK cell immunotherapy alone or in combination with CDDP greatly inhibited tumor growth in hu-BLT mice

**Figure 4:**
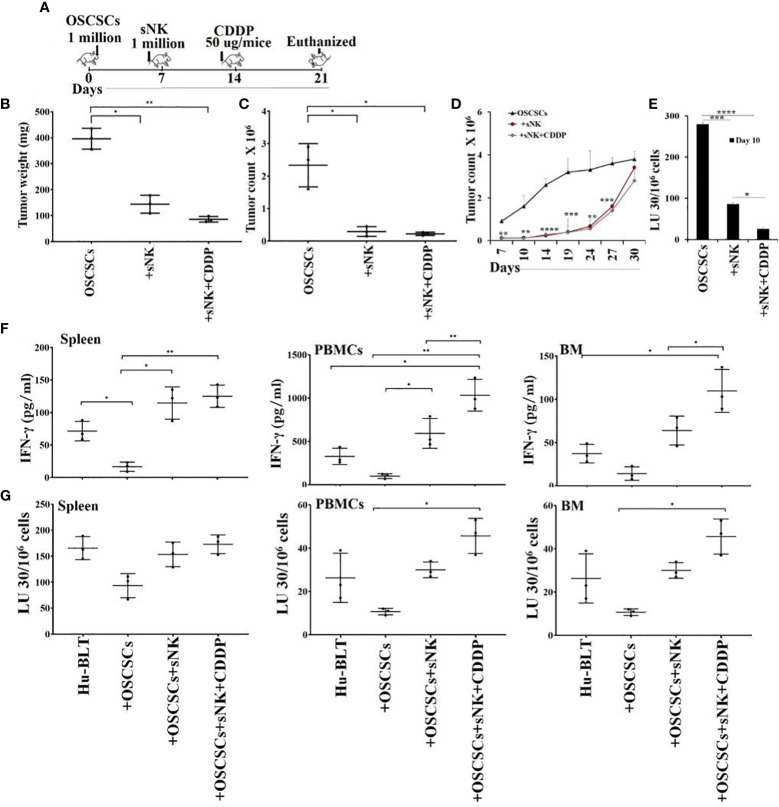
Cisplatin mediated decrease in tumor growth when used sequentially with sNK cell treatment in tumor implanted hu-BLT mice, and increased NK cell-mediated cytotoxicity by immune effectors derived from spleen, bone marrow and peripheral blood. Hu-BLT mice were orthotopically implanted with 1 x 10^6^ human OSCSCs into the floor of the mouth. One week after the tumor implantation, mice received supercharged NK (sNK) cells *via* tail-vein injection, and one week after sNK cell injection, mice received CDDP (50 µg/mice) *via* tail vein injection. The disease progression was monitored for another week **(A)**. Hu-BLT mice were implanted with OSCSC tumors and were injected with sNK cells and CDDP sequentially as depicted in **Figure 4A**. At the end of experiment, hu-BLT mice were sacrificed; the tumors were harvested and weighed (n=3) **(B)**. Hu-BLT mice were implanted with OSCSC tumors and, were injected with sNK cells and CDDP sequentially as depicted in **Figure 4A**. At the end of the experiment, hu-BLT mice were sacrificed; the tumors were harvested, and the single-cells were obtained as described in the Materials and Methods section. Tumor cells were counted microscopically (n=3) **(C)**. Tumor cells were counted microscopically (n=3) **(C)**. Hu-BLT derived tumors were cultured at 1.5 x 10^5^/ ml at the initiation of the cultures. On day 3, unattached cells were removed and fresh media was added. Tumors were detached and counted on days 7, 10, 14, 19, 24, 27, and 30, each time 1 x 10^5^/ ml cells were cultured (n=3) **(D)**. NK cells (1 x 10^6/^ml) from healthy human donors were treated with IL-2 (1000 U/mL) for 18 hours before they were added to ^51^Cr labeled hu-BLT derived tumors at various effector to target ratios. NK-mediated cytotoxicity was determined using 4-hour ^51^Cr release assay. The lytic units (LUs) 30/10^6^cells were determined using inverse number of NK cells required to lyse 30% of the tumor-cells x 100 (n=2 per each experimental condition) **(E)**. Hu-BLT mice were implanted with OSCSC tumors and were injected with sNK cells and CDDP sequentially as shown in **Figure 4A**. At the end of the experiment, hu-BLT mice were sacrificed. Spleens, peripheral blood, and bone marrow were harvested and single cell suspensions were obtained and cultured (1 x 10^6^ /ml) with IL-2 (1000 U/ml) for 7 days. On day 7, the supernatants were harvested and the secretions of IFN-γ were determined using single ELISA (n=3) **(F)**. Spleens, peripheral blood, and bone marrow cells were cultured (1 x 10^6^ /ml) with IL-2 (1000 U/ml) for 7 days. On day 7, cells were used as effectors against OSCSCs using standard 4-hour ^51^Cr release assay. The Lytic units (LU) 30/10^6^ cells were determined using the inverse number of cells required to lyse 30% of OSCSCs x 100 (n=3) **(G)**. ****(p value<0.0001), ***(*p* value 0.0001-0.001), **(*p* value 0.001-0.01), *(*p* value 0.01-0.05).

We used humanized-BLT (hu-BLT) mice model to demonstrate the efficacy of combinational treatment with sNK cells and chemotherapy against human oral CSCs/poorly differentiated tumors. Hu-BLT mice were generated by surgically implanting pieces of human fetal liver and thymus tissues under the renal capsule of NSG mice, followed by tail vein IV injection of same-donor CD34^+^ hematopoietic cells to support full reconstitution of the human bone marrow (38–40). In this study, hu-BLT mice after human immune cell reconstitution were surgically with human OSCSCs in oral cavity followed by IV injections of sNK cells and CDDP sequentially as depicted in Figure (**Figure 4A**). Upon sacrifice, the tumors were harvested and weighed. sNK cells alone or sNK cells combined with CDDP treated mice had smaller tumors in comparison to untreated mice with tumor (**Figure 4B**). Next, we dissociated tumors and recovered single cells to determine tumor cell counts. Tumors from tumor implanted mice treated with sNK cells alone or sNK cells combined with CDDP treated mice had significantly lower numbers of tumor cells as compared to tumor alone implanted mice (**Figure 4C**). When the same numbers of dissociated tumor cells from hu-BLT mice were cultured, significantly lower tumor cell expansion was seen in tumors from sNK cells alone or sNK cells combined with CDDP treated mice as compared to untreated tumors from tumor-bearing mice until day 14-19, after which the tumor growth rate gradually increased and at day 30 the tumor cultures were terminated. Even though the levels of tumor growth approached the levels seen with tumor alone implanted mice, we could still see a higher decrease in tumor growth with sNK+CDDP group as compared to sNK group, and both groups had on average less growth when compared to those from tumor alone implanted mice (**Figure 4D**). When the dissociated tumors of hu-BLT mice were used as targets for primary IL-2 activated NK cells in Cr-release assay, tumors from sNK cells alone or sNK cells combined with CDDP treated mice were killed much less when compared to untreated tumor-bearing mice (**Figure 4E**). In addition, there was statistically significant differences in the resistance of tumor cells to NK cell-mediated cytotoxicity dissociated from sNK+CDDP group as compared to sNK group, and both these groups had highly significant decreases in cytotoxicity when compared to those obtained from tumor alone implanted hu-BLT mice (**Figure 4E**). Our previous studies have demonstrated that CSCs/poorly-differentiated tumors grow at higher rate and are excellent targets of NK cell-mediated cytotoxicity, whereas differentiated tumors grow slow and are resistant to NK cell-mediated cytotoxicity (13, 41–43). Results shown in **Figures 4D, E** indicates that tumors from sNK cells alone or sNK cells combined with CDDP treated mice exhibited characteristics of differentiated tumors. In addition, when all the floating immune cells were removed by changing the media from tumor cultures throughout the days of 7-27 the differentiated nature of tumor cells grown from sNK cells or sNK in combination with CDDP gradually reverted to their CSC/poorly differentiated tumors, and their growth rate gradually increased and approached to those grown from tumor alone implanted BLT mice (**Figures 4D**). We have previously shown that reversion of NK differentiated tumors occurs after two weeks in culture without immune cells, and it correlates with the decreased MHC class I expression on tumor cells (44).

### Sequential treatment with sNK cell immunotherapy and CDDP exhibited increased IFN-γ secretion and NK cell-mediated cytotoxicity by bone marrow, PBMCs and splenocytes of hu-BLT mice

We then assessed IFN-γ secretion and NK cell-mediated cytotoxicity of splenocytes, peripheral blood and bone marrow derived cells of tumor-bearing hu-BLT mice with or without treatments. We observed suppression of both secretion of IFN-γ (**Figures 4F**, **S4**, **S5A**) and NK cell-mediated cytotoxicity (**Figures 4G**, **S5B**) in tumor-bearing untreated mice in comparison to those obtained from healthy mice without tumor implantation. Increase or restoration of IFN-γ secretion and NK cell-mediated cytotoxicity by bone marrow, splenocytes and PBMCs were seen in sNK cell injected tumor-bearing mice, and both these functions were further increased with the combination of sNK cells and CDDP treatment of tumor implanted mice when compared to those with tumor alone implanted mice (**Figures 4F, G**, **S4**, **S5**). CDDP alone injected mice either did not increase or increased slightly the secretion of IFN-γ in cells dissociated from spleen, peripheral blood, and BM (**Figure S4**).

### Differentiated tumors expressed higher levels of PD-L1 and were more susceptible to NK cell-mediated ADCC in the presence of anti-PD-L1 as compared to their stem-like counterparts

**Figure 5:**
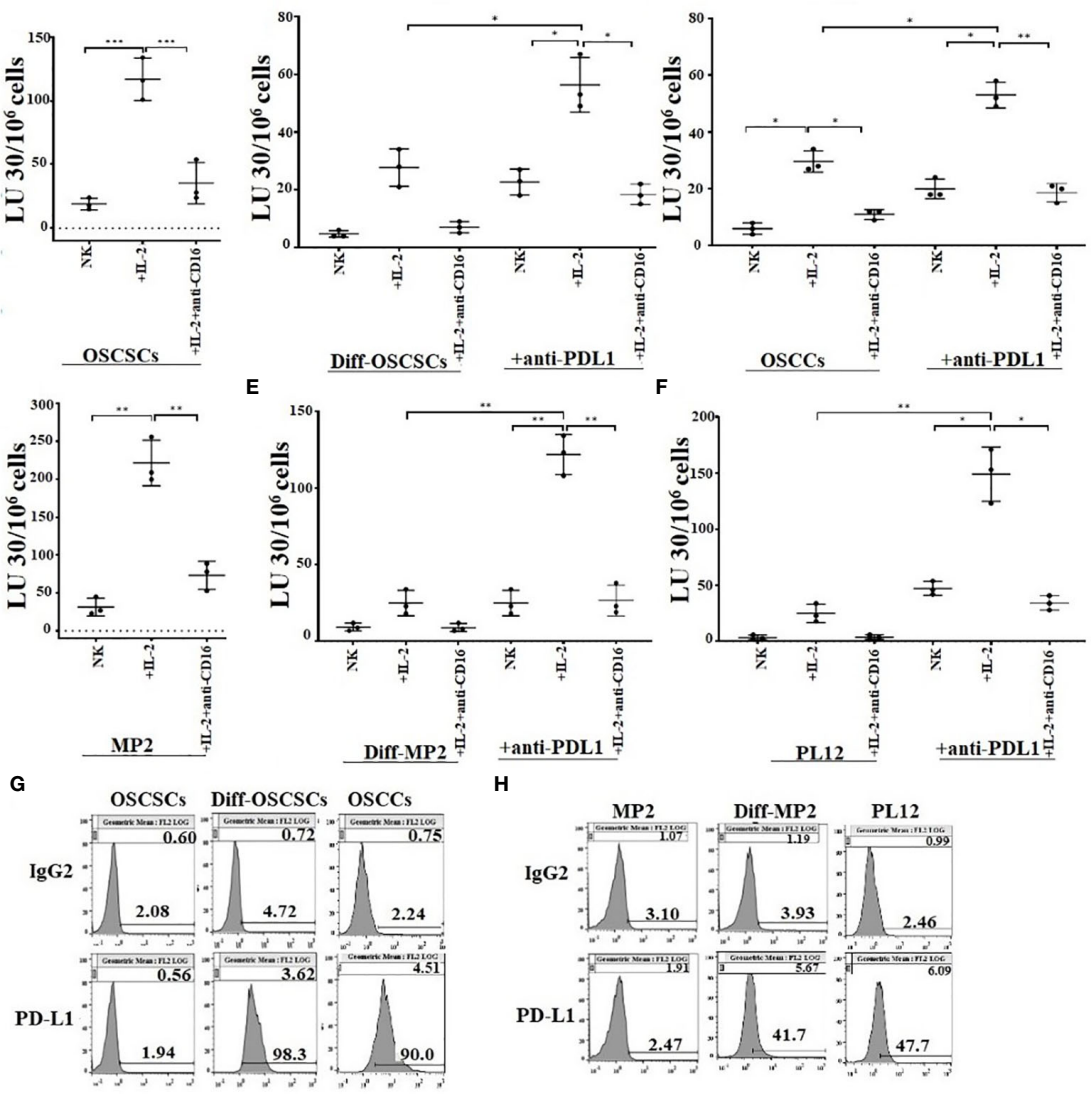
Differentiated tumors expressed higher levels of PD-L1 on their surface and were more susceptible to NK cell-mediated cytotoxicity when compared to their stem-like counterparts in the presence of anti-PDL1. Purified NK cells (1×10^6^ cells/ml) from healthy individuals were left untreated, or treated with IL-2 (1000 U/ml), or treated with IL-2 (1000 U/ml) and anti-CD16 mAbs (3µg/mL) for 18 hours and were used as effectors in chromium release assay. OSCSCs were differentiated using IL-2 (1000 U/ml) and anti-CD16 mAbs (3µg/mL) treated NK cell supernatants as described in Materials and Methods section. OSCSCs **(A)**, NK-differentiated-OSCSCs **(B)**, and differentiated OSCCs **(C)**, were labeled with 51Cr for an hour. NK-differentiated-OSCSCs **(B)**, and differentiated OSCCs **(C)**, 51Cr-labeled tumor cells were then left untreated or treated with anti-PDL1 (5 µg/ml) for 30 minutes. The unbound antibodies were washed away, and the cytotoxicity against the tumor cells was determined using a standard 4-hour 51Cr release assay. The Lytic units (LU) 30/10^6^ cells were determined using the inverse number of NK cells required to lyse 30% of tumors x 100 (n=3) **(A–C)**. The surface expression of PD-L1 was analyzed on tumor cells using flow cytometry. IgG2 isotype control antibodies were used as controls **(G)**. Purified NK cells (1×10^6^ cells/ml) from healthy individuals were left untreated, or treated with IL-2 (1000 U/ml) or treated with IL-2 (1000 U/ml) and anti-CD16 mAbs (3µg/mL) for 18 hours and they were used as effectors in chromium release assay. MP2 cells were differentiated using IL-2 (1000 U/ml) and anti-CD16 mAbs (3µg/mL) treated NK cell supernatants as described in Materials and Methods section. MP2 **(D)**, NK-differentiated MP2 **(E)**, and differentiated PL12 **(F)** were labeled with 51Cr for an hour. NK-differentiated MP2 **(E)**, and differentiated PL12 **(F)** 51Cr-labeled tumor cells were then left untreated or treated with anti-PDL1 (5 µg/ml) for 30 minutes. The unbound antibodies were washed away, and the cytotoxicity against the tumor cells was determined using a standard 4-hour 51Cr release assay. The Lytic units (LU) 30/10^6^ cells were determined using the inverse number of NK cells required to lyse 30% of tumors x 100 (n=3) **(D, E, F)**. The surface expression of PD-L1 was analyzed on tumor cells using flow cytometry. IgG2 isotype control antibodies were used as controls **(H)**. ***(*p* value 0.0001-0.001), **(*p* value 0.001-0.01), *(*p* value 0.01-0.05).

Our previous studies have shown that CSCs/poorly-differentiated tumors are excellent targets of direct NK cell-mediated cytotoxicity, whereas their differentiated counterparts are significantly more resistant (13, 41–43). We have also shown previously that differentiated tumors have higher surface expression of MICA/MICB and are susceptible to ADCC mediated by the primary NK cells in the presence of anti-MICA/MICB antibody, even though NK cells are not able to kill these tumors directly (20). Here, we evaluated NK cell-mediated cytotoxicity against untreated stem-like (OSCSCs and MP2) and untreated or anti-PD-L1 antibody treated differentiated tumors (NK-Diff-OSCSCs, OSCCs, NK-Diff-MP2, and PL12) (**Figure 5A–F**). We demonstrate that NK cells mediated direct cytotoxicity of OSCSCs (**Figure 5A**) and MP2 (**Figure 5D**) tumors, whereas susceptibility to NK cell-mediated cytotoxicity was substantially and significantly lower against NK-diff-OSCSCs (**Figures 5B**, **S6A**), OSCCs (**Figure 5C**, **S6A**), NK-diff-MP2 (**Figures 5E**, **S6B**), and PL12 (**Figure 5F**, **S6B**) tumors when compared to OSCSCs and MP2 cells. NK cell-mediated cytotoxicity was increased against anti-PDL1-treated NK-diff-OSCSCs (**Figure 5B**), OSCCs (**Figure 5C**), NK-diff-MP2 (**Figure 5E**), and PL12 (**Figure 5F**) tumors. In accordance, higher surface expression of PD-L1 was seen on NK-diff-OSCSCs and OSCCs (**Figure 5G**) and NK-diff-MP2 and PL-12 (**Figure 5H**) tumors in comparison to their stem-like counterparts. Differentiated tumors also expressed higher surface expression of MHC-class I (**Figures S7B, C**). We also assessed surface expression of PD-L1 on NK cells (**Figure S7A**). The results indicated that both IL-2 and IL-2+anti-CD16mAb treatment elevated the expression of PD-L1 on NK cells (**Figure S7A**). Taken together, the data indicated that differentiated tumors express higher PD-L1 on their surface, and treatment of these cells with anti-PD-L1 antibody mediate ADCC in the presence of NK cells. However, poorly differentiated tumors are devoid of this surface antigen and therefore NK cells may become inactivated in the presence of anti-PD-L1 antibody treatment since activated NK cells exhibit PD-L1 on the surface.

### Anti-PD1 antibody induced higher IFN-γ secretion from NK cells in the presence of stem-like tumors in comparison to differentiated tumors

**Figure 6:**
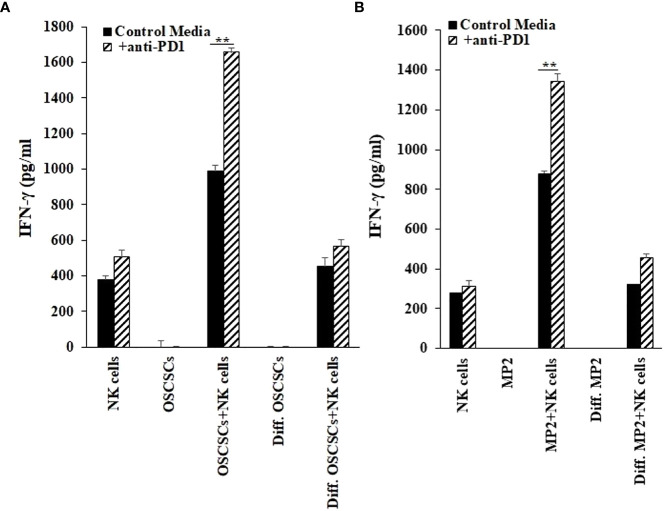
Increased IFN-γ secretion by NK cells in the presence of cancer stem cells and anti-PD1. OSCSCs were differentiated using IL-2 (1000 U/ml) and anti-CD16 mAbs (3µg/mL) treated NK cell supernatants as described in the Materials and Methods section. NK cells of healthy individuals were treated with IL-2 (1000 U/ml) for 18-20 hours before they were co-cultured with tumor cells (NK: tumors; 1:1), and treated with anti-PD1 (500 ng/ml) antibody. On day 3 of co-culture, the supernatants were harvested and the secretion of IFN-γ was determined using single ELISAs **(A)**. MP2 cells were treated with IL-2 (1000 U/ml) and anti-CD16 mAbs (3µg/mL) treated NK cell supernatants to induce differentiation as described in the Materials and Methods section. NK cells of healthy individuals were treated with IL-2 (1000 U/ml) for 18-20 hours before they were added to tumor cells (NK: tumors; 1:1), and were treated with anti-PD1 (500 ng/ml) antibody. On day 3 of co-culture, the supernatants were harvested and the secretion of IFN-γ was determined using single ELISA **(B)**. **(*p* value 0.001-0.01).

We have previously demonstrated that NK cells secrete higher levels of IFN-γ when co-cultured with CSCs/poorly differentiated tumors in comparison to differentiated tumors (42). In our current study, we co-cultured stem-like/poorly differentiated (MP2 and OSCSCs) and their differentiated counterparts (NK-Diff-MP2 and NK-Diff-OSCSCs) with NK cells with or without anti-PD1 treatment (**Figures 6**, **S7**). Lower secretion of IFN-γ was found when NK cells were co-cultured with NK-diff-OSCSCs (**Figures 6A**, **S8**) and NK-diff-MP2 (**Figure 6B**) in comparison to the cultures with their stem-like counterparts. Anti-PD1 treated NK cells without tumors showed slightly increased levels of IFN-γ secretion, however, the highest effect of anti-PD1 treatment was seen when NK cells were co-cultured with OSCSCs (**Figures 6A**, **S8**) or MP2 (**Figure 6B**) cells in comparison to those co-cultured with their NK-differentiated counterparts.

### sNK cell immunotherapy alone or in combination with anti-PD1 antibody inhibited tumor growth in hu-BLT mice and significantly improved immune function of hu-BLT mice

**Figure 7:**
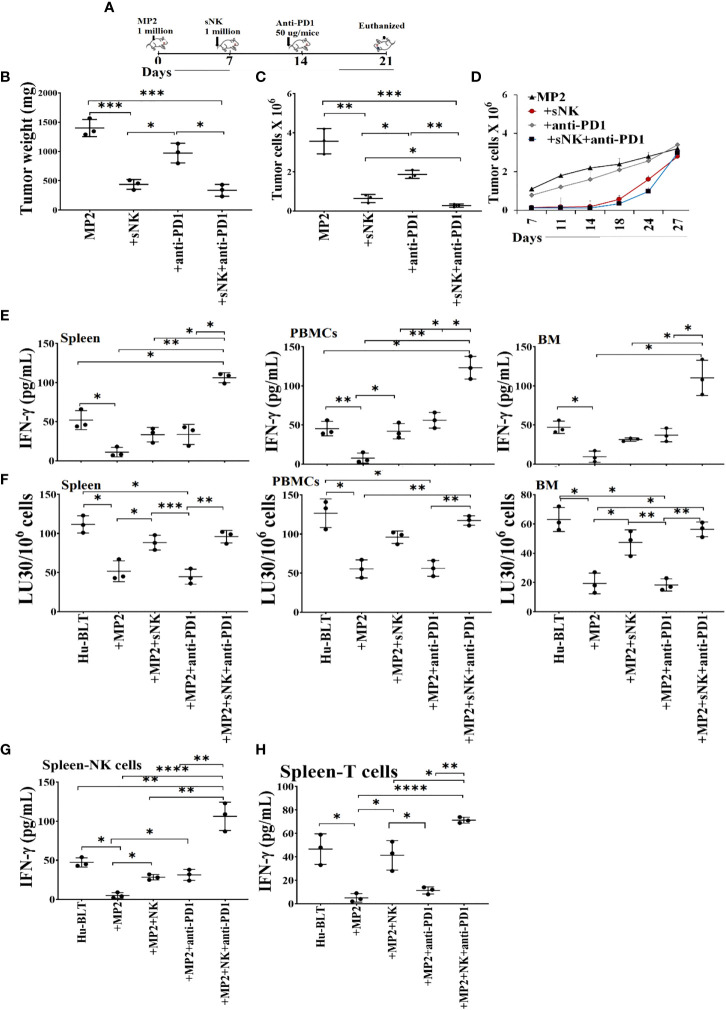
Combination of sNK cells and anti-PD1 antibody halted the growth of MP2 tumors, and increased IFN-γ secretion and cytotoxic function by PBMCs, splenocytes and bone marrow derived immune cells from hu-BLT mice. Hu-BLT mice were orthotopically injected with 1 x 10^6^ human MP2 tumors in the pancreas. One week after the tumor implantation, mice received supercharged NK (sNK) cells *via* tail-vein injection, and one week after sNK cell injection, mice received anti-PD1 (50 µg/mice) antibody *via* tail vein. The disease progression was monitored for another week **(A)**. Hu-BLT mice were implanted with MP2 tumors and were injected with sNK cells and anti-PD1 antibody as depicted in **Figure 7A**. At the end of the experiment, hu-BLT mice were sacrificed and the tumors were harvested and weighed (n=3) **(B)**. Hu-BLT mice were implanted with MP2 tumors and were injected with sNK cells and anti-PD1 antibody as depicted in **Figure 7A**. At the end of the experiment, hu-BLT mice were sacrificed and the tumors were harvested and single-cells were isolated as described in Materials and Methods section. Tumor cells were counted microscopically (n=3) **(C)**. Hu-BLT derived tumors were cultured at 0.15 x 10^6^/ml at the initiation of the tumor cultures, and the cell growth were determined on the days shown in the Figure. Statistical analysis is shown for sNK or sNK+anti-PD1 antibody group vs. untreated group (n=3) **(D)**. Hu-BLT mice were implanted with MP2 tumors and, were injected with sNK cells and anti-PD1 antibody as depicted in **Figure 7A**. At the end of the experiment, hu-BLT mice were sacrificed and the spleens, peripheral blood, and bone marrow were harvested, and single cell suspensions were prepared and cultured in the presence of IL-2 (1000 U/ml) for 7 days. On day 7, the supernatants were harvested and the secretion of IFN-γ was determined using single ELISA (n=3) **(E)**. Splenocytes, and peripheral blood and bone marrow derived immune cells were cultured in the presence of IL-2 (1000 U/ml) for 7 days. On day 7, cells were used as effectors against OSCSCs using standard 4-hour ^51^Cr release assay. The Lytic units (LU) 30/10^6^ cells were determined using the inverse number of cells required to lyse 30% of OSCSCs x 100 (n=3) **(F)**. NK **(G)** and CD3+ T **(H)** cells were purified from the spleen and cultured in the presence of IL-2 (1000 U/ml for NK cultures; and100 U/ml for T cell cultures) for 7 days. On day 7, the supernatants were harvested and the secretion of IFN-γ was determined using single ELISA (n=3) **(G, H)**. ****(p value<0.0001), ***(*p* value 0.0001-0.001), **(*p* value 0.001-0.01), *(*p* value 0.01-0.05).

Hu-BLT mice were surgically implanted with human MP2 tumors followed by injections of sNK cells and anti-PD1 sequentially as depicted in **Figure 7A**. Upon sacrifice, the tumors were harvested and weighed, and the results were compared to tumor bearing mice in the absence of treatments. sNK cells alone or sNK cells combined with anti-PD1 antibody injected mice had smaller tumors in comparison to untreated tumor implanted mice (**Figures 7B**, **S9A**). Anti-PD1 antibody alone treated mice had smaller tumors compared to untreated tumor-bearing mice but the size was larger in comparison to either sNK treated or sNK + anti-PD1 antibody treated group (**Figure 7B**). Tumors were then dissociated and counted and the numbers were adjusted to 1.5X10^5^ per well. Tumor growth were slightly with anti-PD1 alone treatment, and was much less with sNK cells alone or sNK cells combined with anti-PD1 treatments when compared to untreated tumor implanted mice (**Figures 7C**). When the same numbers of dissociated tumor cells from hu-BLT mice were cultured, significantly lower tumor cell expansion was seen in tumors from sNK cells alone or sNK cells combined with anti-PD-1 antibody treated mice as compared to those from untreated tumor-bearing mice until day 14-18, after which the tumor growth rate gradually increased and approached to those obtained from tumor alone implanted mice, and at day 27 the tumor cultures were terminated. Even though the levels of tumor growth gradually approached the levels seen with tumor alone implanted mice at the days 18-24, we could still see a lower tumor growth with sNK+anti-PD-1 antibody treated group as compared to sNK treated group, and both groups had on average lower growth when compared to those from tumor alone implanted mice (**Figures 7D**, **S9B**). Tumors dissociated from anti-PD-1 antibody group exhibited a lower rate of tumor growth when compared to the tumor alone implanted group but the levels of tumor growth were higher than those obtained from either the sNK treated or the sNK+anti-PD-1 antibody treated group (**Figures 7D**, **S8B**). In addition, when all the floating immune cells were removed by changing the media every three days from tumor cultures throughout the days of 7-27 the differentiated nature of tumor cells grown from sNK cells or sNK in combination with anti-PD-1 gradually reverted to their CSC/poorly differentiated tumors, and their growth rate gradually increased and approached to those grown from tumor alone implanted BLT mice (**Figure 7D**).

### Sequential treatment with sNK cells and anti-PD1 augmented IFN-γ secretion and NK cell-mediated cytotoxicity by immune cells of spleen, peripheral blood, and bone marrow of tumor bearing hu-BLT mice

We observed suppression of both secretion of IFN-γ (**Figures 7E, G, H**, **S10A**, **S11A**) and NK cell-mediated cytotoxicity (**Figures 7F**, **S10B**, **S11B**) in tumor-bearing hu-BLT mice in comparison to those from healthy mice. Restoration or increased IFN-γ secretion and NK cell-mediated cytotoxicity were seen in tumor-bearing mice injected with sNK cells alone or anti-PD1 alone, and both of these functions were further increased with the combination of sNK cells and anti-PD1 antibody injection in tumor bearing mice (**Figures 7E–H**, **S9**, **S10**). It is important to note that the levels of IFN-γ secretion were comparable between sNK cells treated mice and anti-PD-1 treated mice; however, the NK cell mediated cytotoxicity was much higher in sNK treated tumor bearing mice than in the presence of anti-PD-1 treated tumor bearing mice (**Figures 7E–H**, **S10**, **S11**). The combination of both sNK and anti-PD-1 treatment significantly elevated IFN-γ secretion when compared to each treatment alone and increased cytotoxicity more than those seen in the presence of sNK treatment alone (**Figures 7E–H**, **S10**, **S11**). Taken together, these results indicated that sequential treatment of sNK cells with anti-PD-1 antibody is capable of increasing cytotoxic function in tumor-implanted hu-BLT mice and significantly augmented the secreted IFN-γ in immune cells from bone marrow, spleen and peripheral blood.

## Discussion

NK cells are indispensable for the treatment of cancer due to their many important functions. We have come a long way in understanding the mechanisms underlying activation and increased function of NK cells, however, we still do not have a cure or even successful treatment for aggressive cancers. Most problems stem from not having the full understanding of NK function in cancer patients, and many underlying mechanisms of NK cell function still await clarifications. The mere fact that NK cells are specialized to target CSCs/poorly differentiated aggressive tumors, should place these cells at the top of any treatment strategies. Indeed, the function of NK cells found to be compromised in many if not all cancer patients provide the rationale for the induction and progression of cancer since NK cells eliminate the clones that seed the cancer (28, 45, 46). In addition, loss of NK cell function at the preneoplastic stage of tumorigenesis before the establishment of pancreatic cancer is a testament to the crucial roles that NK cells play in suppression of cancer (47, 48). Moreover, NK cells differentiate tumors through the production of IFN-γ and TNF-α leading to decreased expansion and progression of cancer, in addition to the conditioning of T cells to target tumor cells. In this regard, sNK cells with exceptional expansion and functional capabilities, select CD8+ T cells and expand their numbers and function allowing the formation of memory/effector cells (45). Indeed, successful eradication of cancer is partly dependent on formation of tumor specific memory/effector CD8+ T cells, and tumors infiltrated by CD8+ T cells were shown to have a much better prognosis in patients (49). When comparing the function of primary IL-2 activated NK cells or IL-2+anti-CD16mAb or IL-2+anti-CD16mAb+sAJ2 treated NK cells with sNK cells, many significant differences could be seen on genetic as well as protein and functional levels (**Figure 1** and manuscript in prep) (28). Briefly, sNK cells are a unique population of NK cells with completely distinct profiles from those of untreated and IL-2 treated NK cells at the RNA seq analysis at the single cell level based on UMAP and regulon profiles, and in terms of cell cycle analysis, granule content and functional capabilities (**Figure 2**). When comparing to primary NK cells treated with different treatment strategies (**Figure 1A**) or those cultured with K562 or OSCSCs or MP2 or PBMCs (**Figure S1**), sNK cells proliferated up to 27-34 days, mediated much higher cytotoxicity and secreted much higher levels of IFN-γ whereas the primary NK cells after day 6 of culture lost their expansion capability and had minimal function (**Figure 1**). Having generated such a unique and potent population of NK cells, we aimed at understanding their effect in combination with other therapeutic modalities. Furthermore, by better understanding sNK cell function, we were able to establish combinatorial therapies to successfully treat cancer in hu-BLT mice. In this regard, we demonstrated two different treatments that can be combined with sNK cells not only restore or further activate the NK cells but also provide a strategy to augment the efficacy of the treatment with other treatment modalities to successfully eradicate all different subpopulations of tumor cells in the tumor microenvironment. In this case, we have previously shown that NK cell-differentiated tumors become susceptible to chemotherapeutic drugs (12). Indeed, treatment of tumor cells with supernatants from sNK cells not only increased the differentiation antigens such as MHC-class I, CD54, PDL-1 but it also curtailed their growth and made the tumors susceptible to chemotherapeutic drugs in *in vitro* experiments published previously and shown in here (12) (**Figures 3**, **S3**, **S6**). Furthermore, combining sNK cell treatment with chemotherapy drugs augmented the targetability of tumor cells by the chemotherapeutic drugs *in vivo*, as evidenced by the *in vitro* data. To validate our *in vitro* observations reported previously and in here, and to test the premise that sNK cells treated tumors become targetable by the chemotherapeutic drugs *in vivo*, we performed experiments in hu-BLT mice by first targeting and differentiating tumors with sNK cells (**Figure 4**) (12) followed by the use of chemotherapy drugs to target the differentiated tumors. In this paper, we showed that one dose of 1X10^6^ sNK cell injection not only kills but also differentiates tumors in tumor bearing hu-BLT mice, allowing chemotherapy drugs to target the remaining tumors, thereby decreasing the tumor load, and also augmenting the secretion of IFN-γ by the NK cells from humanized mice. Such combinatorial treatments will establish a circular pattern in which sNK cells will increase the effectiveness of chemotherapeutic drugs in targeting tumors but also the chemotherapy drugs will increase the function of sNK cells to target more tumors. Thus, these treatment strategies should be able to remove the heterogenous nature of tumor cells, allowing restoration of NK cell function in cancer patients to prevent cancer recurrences. When tumors were resected and single cells were prepared and cultured, tumors from OSCSC implanted mice grew and proliferated at a much higher rate than those cultured either from sNK injected or sNK+CDDP treated tumor implanted mice (**Figure 4D**). Tumor growth was much less in sNK and sNK+CDDP treated tumor implanted mice until day 24 after which they started to increase their growth potential and the growth rate became closer to the tumors resected from tumor alone implanted mice. Coincided with increase in tumor growth was the decrease in MHC-class I expression on the tumor cells since the differentiated tumors were not supplied by either sNK cells or their supernatants, therefore, the tumors reverted to the poorly differentiated/CSC stage at the end of cultures. The reversion could be due to de-differentiation of the tumors or selection of tumors with CSCs/poorly differentiated phenotype which has much lower MHC class I expression. Indeed, in our previous paper we established that all the tumors initially exhibited differentiated phenotype and later lost the differentiation antigens and became poorly differentiated tumors (12, 13). Thus, those results argued for the de-differentiation of tumors rather than selection (13). In addition, when day 10 tumor cultures were tested in cytotoxicity against fresh IL-2 activated primary NK cells, those that were obtained from tumor implanted and sNK or sNK+CDDP injected mice had much lower susceptibility to NK cell mediated cytotoxicity when compared to those cultured from tumor alone implanted mice, indicating the increased differentiation and acquisition of MHC-class I antigens in these cultures. Interestingly, in this assay we could see significant differences in the decreases of cytotoxicity between sNK and sNK+CDDP tumor cultures, indicating higher differentiation stage of sNK+CDDP tumor cultures as compared to sNK tumor cultures (**Figure 4E**) (13, 41–43). In agreement with our studies, previous work from other labs demonstrated cisplatin mediated up-regulation of NK cell cytotoxicity through suppression of AR, and upregulation of ULBP-2 in the HCC tumor model (50). In addition, Low-dose cisplatin administration prevented suppression of NK cell activity in patients with gastrointestinal cancer (51). Finally, the use of combination of cisplatin and natural killer cells overcame cisplatin resistance in ovarian cancer (52).

Check-point inhibitors such as anti-PD-1 and anti-PD-L1 are becoming standard of care for many cancers; however, even though they work for certain cancer types and in certain cancer patients, not all cancer patients benefit from such treatments. To increase the effectiveness of both NK cells and anti-PD-1 therapy we sequentially we sequentially treated the tumor bearing hu-BLT mice with sNK cells and anti-PD-1 therapy and found such treatment to not only prevent and remove most of tumors from the mice but also it augmented the function of immune cells by increasing the secretion of IFN-γ when both treatments were used in mice. Indeed, anti-PD-1 treatment of NK cells in the presence of CSC/poorly differentiated tumors augmented the secretion of IFN-γ by the NK cells, indicating that NK cells are capable of activation through PD-1 surface receptors similar to those of T cells (**Figures 6**, **7G**, **S7**). Indeed, sequential treatment of tumor bearing hu-BLT mice with sNK cells and anti-PD-1 antibody increased the release in IFN-γ by the immune effectors notably both the NK cells and T cells and halt the tumor growth and expansion (**Figures 7G, H**). Similar to the *in vivo* experiments with sNK+CDDP treatment, when tumors were resected from the sNK+anti-PD-1 treated mice, and single cells were prepared and cultured, tumors from MP2 implanted mice grew and proliferated at a much higher rate than those cultured either from sNK injected or sNK+anti-PD-1 treated and tumor implanted mice (**Figure 7D**). Tumor growth was much less in sNK and sNK+ anti-PD-1 treated and tumor implanted mice until day 18-24, after which they started to increase their growth potential and the growth rate became closer to the tumors resected from tumor alone implanted mice at day 27. Coinciding with the increase in tumor growth was the decrease in MHC-class I expression on the tumor cells since the differentiated tumors were not supplied by either sNK cells or their supernatants, therefore, the tumors reverted to the poorly differentiated/CSC stage at the end of the cultures. The reversion could be due to de-differentiation of the tumors or selection of tumors with poorly differentiated/CSC phenotype as stated above. In accordance with our studies, adoptive transfer of *ex vivo* IL-2-activated NK cells combined with anti-PD-1 resulted in tumor growth inhibition in a xenograft gastric cancer model (53). In another study, PD-1 and PD-L1 blockade induced a strong NK cell response that was found to be indispensable for the full therapeutic effect of immunotherapy (54). In addition, the authors showed that PD-1 was expressed on NK cells within transplantable, spontaneous, and genetically induced mouse tumor models. Furthermore, PD-1 expression was higher on NK cells with a more activated phenotype with no evidence of exhausted phenotype.

However, one has to take precaution in interpreting the *in vivo* data because the heterogeneity of tumor cells in terms of their differentiation stage may make the results very difficult to interpret. This could be one reason why certain cancer patients are able to benefit from the check-point inhibitors and yet others do not. For instance, the use of anti-PD-L1 antibody can have completely different effect on NK cells depending on the stage of differentiation of tumor cells, as seen in our study (**Figure 5**). If competent NK cells have infiltrated tumors with a higher fraction of CSCs/poorly differentiated tumors, they should be able to eliminate these tumors in direct cytotoxicity (**Figure 5**). In addition, higher expression of PD-L1 on tumor-activated NK cells may make NK cells themselves to become susceptible to ADCC, and decrease in the cytotoxic function of NK cells. On the other hand, if the tumor phenotype is tilted towards a well-differentiated phenotype, this may increase effectiveness of NK cells in mediating ADCC since tumor cells will be upregulating PD-L1 and becoming susceptible to NK cell-mediated ADCC effect, whereas such tumors are not, or are less susceptible to direct cytotoxicity by the primary NK cells as seen in our studies (**Figure 5**). Therefore, when such therapies fail in patients, one has to not only understand the nature of NK cells but also what type of tumors NK cells are targeting.

Finally, in this paper we present two different combinatorial therapies that will likely be successful in patients. There are many others such as combination of NK cells with CD8+ T cells, NK cells with radiotherapy, NK cells with virotherapy, NK cells with bacterial therapy, etc. All of these different scenarios are under investigation in our laboratory and should provide exciting treatment strategies for cancer therapy in the future.

## Materials and methods

### Cell lines, reagents, and antibodies

Oral squamous carcinoma stem cells (OSCSCs) and oral squamous cell carcinoma (OSCCs) were isolated from patients with tongue tumors at UCLA (13, 42, 55). NK cells, OSCSCs, and OSCCs were cultured in RPMI 1640 (Invitrogen by Life Technologies, CA), supplemented with 10% fetal bovine serum (FBS) (Gemini Bio-Products, CA). Recombinant IL-2 was obtained from NIH-BRB. Antibodies to CD16 were purchased from Biolegend (San Diego, CA). Antibodies used for flow cytometry – IgG2, MHC-class I, and B7H1 (PD-L1) were purchased from Biolegend (San Diego, CA). MIA PaCA-2 (MP2), PL12, and Capan human pancreatic cancer cell lines were provided by Dr. Nicholas Cacalano (UCLA, School of Medicine, CA, USA), and were cultured in DMEM supplemented with 10% FBS. Cisplatin and Paclitaxel were purchased from Ronald Reagan Pharmacy at UCLA. ELISA kits for IFN-γ were purchased from Biolegend (San Diego, CA), and multiplex analysis kit was purchased from Millipore (Billerica, MA). Propidium iodide (PI) and chromium-51 was purchased from PeproTech (Cranbury, NJ, USA) and Perken Elmer (Waltham, MA, USA), respectively. Chromium Single cell 3’ Reagent kit v3, Cat#1000075 was purchased from10X Genomics (Pleasanton, CA, USA).

### Purification of human NK cells and monocytes

Written informed consents, approved by UCLA Institutional Review Board (IRB), were obtained from healthy individuals, and all procedures were approved by the UCLA-IRB. Peripheral blood was separated using ficoll-hypaque centrifugation, after which the white layer, containing peripheral blood mononuclear cells (PBMCs) was harvested. NK cells and monocytes were negatively selected from PBMCs using the EasySep^®^ Human NK cell enrichment and EasySep^®^ Human monocytes enrichments kits, respectively, purchased from Stem Cell Technologies (Vancouver, BC, Canada). Purified NK cells and monocytes were stained with anti-CD16 and anti-CD14, respectively, to measure purity using flow cytometric analysis. Samples showing greater than 95% purity were used for the study.

### NK cell supernatant collection and stem cell differentiation

Purified NK cells were activated with rh-IL-2 (1000 U/ml) and anti-CD16 mAb (3 µg/ml) for 18-20 hours before the supernatant was harvested, and was used in differentiation of OSCSCs, and MP2 cells. The supernatant volume was determined based on IFN-γ required, and was accessed by ELISA specific to IFN-γ. Differentiation of OSCSCs and MP2 cells were conducted with an average total amount of 2000 pg and 5000 pg, respectively, over the course of 5 days. On day 0, 1 × 10^6^ tumor cells were cultured, on day 1 unattached tumor cells were removed and attached tumor cells were treated with NK cell supernatants on days 1, 2, 3 and 4. On day 5, tumor cells were rinsed with 1 X PBS, detached and used for experiments.

### Generation of osteoclasts and supercharged NK cells

To generate osteoclasts (OCs), monocytes were cultured in alpha-MEM media supplemented with M-CSF (25 ng/mL) and RANKL (25 ng/mL) for 21 days, media was replenished every three days. Human purified NK cells were activated with rh-IL-2 (1000 U/ml) and anti-CD16 mAb (3 µg/ml) for 18-20 hours before they were co-cultured with OCs and sAJ2 (OCs : NK:sAJ2; 1:2:4) in RPMI 1640 medium containing 10% FBS. Probiotic bacteria, AJ2 is a combination of seven to eight different strains of gram-positive probiotic bacteria (*Streptococcus thermophiles, Bifidobacterium longum, Bifidobacterium breve, Bifidobacterium infantis, Lactobacillus acidophilus, Lactobacillus plantarum, Lactobacillus casei, and Lactobacillus bulgaricus*) selected for their superior ability to induce optimal secretion of both pro-inflammatory and anti-inflammatory cytokines in NK cells (32). The medium was refreshed every three days with RPMI containing rh-IL-2 (1500 U/ml).

### Western blot analysis

Cells were washed twice with an ice-cold PBS and lysed in NP-40 lysis buffer supplemented with protease inhibitor cocktail (APExBIO). Lysates were centrifuged at 16000g at 4°C for 20 minutes to obtain post-nuclear cell fraction. Protein concentration was determined with Pierce BCA protein assay kit (ThermoFisher Scientific). Non-reducing SDS-PAGE was performed, and separated proteins were transferred to nitrocellulose membrane. Membranes were blocked for 1 hour in 5% non-fat dry milk in PBS. Membranes were incubated in primary antibodies overnight at 4°C and HRP conjugated secondary antibodies for 1h at room temperature. Bands were visualized with Clarity Max Western ECL substrate (BioRad). Images were acquired with ChemiDoc ML imaging System (Biorad). The following primary antibodies were used: mouse anti-granzyme B (sc-8022, Santa Cruz Biotechnology), mouse anti-cathepsin C (sc-74590, Santa Cruz Biotechnology), mouse anti-perforin-1 (sc-136994, Santa Cruz Biotechnology). We used anti-mouse HRP conjugated secondary antibodies (405306 BioLegend). Stain free technology (BioRad) was used for loading control.

### Single-cell RNA sequencing

Single-cell RNA sequencing was performed using a 10X Chromium machine. Single-cell cDNA libraries were prepared using the 10X Chromium Single cell 3’ Reagent kit v3 and sequenced *via* Illumina Novaseq 6000 (Illumina) to a depth of around 30 thousand reads per cell. Raw data from each sample were demultiplexed and aligned to a custom reference genome (GRCh38), and UMI counts were quantified using 10X Genomics CellRanger software (v3.0.0) with default parameters. Single-cell clustering and cell-cycle scoring are performed using the Seurat package (v3.0). Single-cell regulatory network inference and clustering (SCENIC) is done by using the SCENIC R package (1.2.0) with the hg38 database (https://resources.aertslab.org/cistarget/).

### Tumor implantation in hu-BLT mice

Animal research was performed under the written approval of the UCLA Animal Research Committee (ARC) in accordance with all federal, state, and local guidelines. Combined immunodeficient NOD.CB17-Prkdcscid/J and NOD.Cg-Prkdcscid Il2rgtm1Wjl/SzJ (NSG lacking T, B, and NK cells) were purchased from Jackson Laboratory. Humanized-BLT (hu-BLT; human bone marrow/liver/thymus) mice were prepared on NSG background as previously described (38, 56). To establish orthotopic tumors, mice were first anesthetized with isoflurane in combination with oxygen, and 1 x 10^6^ human OSCSCs and MP2 tumor cells suspended in 10 μl HC Matrigel were then injected directly into the floor of their mouths and pancreas, respectively. One week after tumor implantation mice received 1 x 10^6^ OC-expanded supercharged NK cells *via* tail vein injection. One week after NK injections, mice received CDDP (50 µg/mice) or anti-PD1 (50 µg/mice) *via* tail vein injection. One week later, mice were euthanized, and tumors, bone marrow, spleen, and peripheral blood were harvested.

### Cell isolation and cell cultures of tumors and immune cells of hu-BLT mice

The oral and pancreatic tumors harvested from hu-BLT mice were immediately cut into 1 mm^3^ pieces and placed into a digestion buffer containing 1 mg/ml collagenase II (oral tumor) or collagenase IV (pancreatic tumor), 10 U/ml DNAse I, and 1% bovine serum albumin (BSA) in DMEM media, and incubated for 20 minutes at 37°C oven on a 150 rpm shaker. After digestion, the sample were filtered through 40 mm cell strainer and centrifuged at 1500 rpm for 10 minutes at 4°C. The pellet was re-suspended in DMEM media and cells were counted. To obtain single-cell suspensions from BM, femurs were cut at both ends and flushed by using RPMI 1640 media; afterwards, BM cells were filtered through a 40 µm cell strainer. To obtain single-cell suspensions from spleen, the spleens were minced, and the samples were filtered through a 40 µm cell strainer and centrifuged at 1500 rpm for 5 minutes at 4°C. The pellets were re-suspended in ACK buffer for 2-5 mins to remove the red blood cells followed by re-suspension in RPMI media and centrifugation at 1500 rpm for 5 minutes at 4°C. PBMCs were isolated from peripheral blood using Ficoll-Hypaque centrifugation of heparinized blood specimens. The buffy coats containing PBMCs were harvested, washed, and re-suspended in RPMI 1640 medium. Cells obtained from each tissue sample were treated with IL-2 (1000 U/ml) and cultured in RPMI 1640 medium containing 10% FBS for 7 days.

### Surface staining and cell death analysis

Staining was performed by labeling the cells with antibodies as described previously (43, 57, 58). The percentage of dead cells was determined by propidium iodine (PI) (100 μg/ml) staining using flow cytometric analysis. Flow cytometric analysis was performed using Attune NxT flow cytometer (Thermo Fisher Scientific, Waltham, MA) and FlowJo v10.4 (BD, Oregon, USA) were used for analysis. For selected experiments Beckman Coulter Epics XL cytometer (Brea, CA) was also used, and the results were analyzed in the FlowJo vX software (Ashland, OR).

### Enzyme-linked immunosorbent assays and multiplex cytokine assay

Single ELISAs were performed as previously described (43). To analyze and obtain the cytokine and chemokine concentration, a standard curve was generated by either two- or three-fold dilutions of recombinant cytokines provided by the manufacturer. For multiple cytokine array, the levels of cytokines were determined by multiplex assay, which was conducted as described in the manufacturer’s protocol for each specified kit. Analysis was performed using a Luminex multiplex instrument (MAGPIX, Millipore, Billerica, MA), and data was analyzed using the proprietary software (xPONENT 4.2, Millipore, Billerica, MA).

### ^51^Cr release cytotoxicity assay

The ^51^Cr release cytotoxicity assay was performed as previously described (59). Briefly, different ratios of effectors and ^51^Cr–labeled target cells were incubated for four hours. After which, the supernatants were harvested from each sample, and the released radioactivity was counted using the gamma counter. The percentage specific cytotoxicity was calculated as follows:


$$\text{\%cytotoxicity}=\frac{\text{Experimental cpm}-\text{spontaneous cpm}}{\text{Total cpm}-\text{spontaneous cpm}}$$

LU 30/10^6^ was calculated by using the inverse of the number of effector cells needed to lyse 30% of target cells ×100.

### Statistical analyses

Prism-9 software was used for statistical analysis. An unpaired or paired, two-tailed Student’s t-test was performed for experiments with two groups. One-way ANOVA with a Bonferroni post-test was used to compare different groups for experiments with more than two groups. Duplicate or triplicate samples were used for assessment. The following symbols represent the levels of statistical significance within each analysis: ****(p value<0.0001), ***(*p* value 0.0001-0.001), **(*p* value 0.001-0.01), *(*p* value 0.01-0.05).

## Data availability statement

The data presented in the study are deposited in the NCBI GEO repository, accession number GSE226160.

## Ethics statement

The studies involving human participants were reviewed and approved by UCLA Institutional Review Board (IRB). The patients/participants provided their written informed consent to participate in this study. The animal study was reviewed and approved by UCLA Animal Research Committee (ARC).

## Author contributions

KK generated and analyzed data, wrote, reviewed and edited the manuscript. P-CC, M-WK, AM, ES generated supporting data and edited manuscript. SM, JK reviewed and edited manuscript. AJ oversaw the studies, conceptualization of the report, reviewed and edited the report, and acquired funding. All authors contributed to the article and approved the submitted version.
