# Supplementary material for: Sequential therapy with supercharged NK cells with either chemotherapy drug cisplatin or anti-PD-1 antibody decreases the tumor size and significantly enhances the NK function in Hu-BLT mice
Source: Front Immunol. 2023 May 1;14:1132807. doi: 10.3389/fimmu.2023.1132807 (PMC10183580; doi:10.3389/fimmu.2023.1132807)
Supplement: Supplementary file 1 [file DataSheet_1.docx]

**Supplementary Data**

**Figure S1: OC induced higher cell expansion and functional activation of NK cells in comparison to K562, OSCSCs, OSCCs, PBMCs and MP2 cells**

NK cells (1 x 10^6^ cells/ml) from healthy individuals were treated with a combination of IL-2 (1000 U/ml), or IL-2 (1000 U/ml)+anti-CD16mAbs (3 µg/ml), or IL-2 (1000 U/ml)+sAJ2 (NK:sAJ2; 1:2), or IL-2 (1000 U/ml)+anti-CD16mAbs (3 µg/ml)+sAJ2 (NK:sAJ2; 1:2) or IL-2 (1000 U/ml)+sAJ2+OCs (OCs:NK:sAJ2; 1:2:4), or IL-2 (1000 U/ml)+anti-CD16mAbs (3 µg/ml)+sAJ2+OCs (OCs:NK:sAJ2; 1:2:4), or IL-2 (1000 U/ml)+sAJ2+K562 (K562:NK:sAJ2; 1:2:4), or IL-2 (1000 U/ml)+anti-CD16mAbs (3 µg/ml)+sAJ2+K562 (K562:NK:sAJ2; 1:2:4), and cells were counts on days shown on Figure. NK cell-mediated cytotoxicity against oral squamous cell carcinoma stem cell line (OSCSCs) was determined on the day 8 using a standard 4-hour ^51^Cr release assay. The lytic units 30/10^6^ cells were determined using the inverse number of NK cells required to lyse 30% of OSCSCs x 100 **(A)**. NK cells were cultured as described in Fig. S2A using OCs, K562, irr-OCs and irr-K562 as feeder cells, the supernatants were harvested on days 6 and 7 to determine IFN-γ secretion using single ELISA, and NK cell-mediated cytotoxicity against OSCSCs was determined on the day 6 using a standard 4-hour ^51^Cr release assay. The lytic units 30/10^6^ cells were determined using the inverse number of NK cells required to lyse 30% of OSCSCs x 100 **(B)**. NK cells were cultured as described in Fig. S2A using OCs, and OSCSCs as feeder cells, cells were counted and NK cell-mediated cytotoxicity against OSCSCs was determined on the day 8, the supernatants were harvested on days 1,3,6 and 8 to determine IFN-γ secretion using single ELISA **(C)**. NK cells were cultured as described in Fig. S2A using OCs, irr-OSCC, irr-OSCSCs, irr-PL12, and irr-MP2 feeder cells, and cells were counted, and the supernatants were harvested on day 8 to determine IFN-γ secretion using single ELISA **(D)**. NK cells were cultured as described in Fig. S2A using OCs, monocytes and irr-PBMCs feeder cells, and the supernatants were harvested on days 3, 9, 12, and 15 to determine IFN-γ secretion using single ELISA **(E)**.

**Figure S2: OC-expanded supercharged NK cells exhibit higher cytotoxic granules, and secretion of IFN-γ in comparison to primary NK cells.**

Osteoclasts (OCs) were generated as described in Materials and Methods section. NK cells (1 x 10^6^ cells/ml) from healthy individuals were treated with a combination of IL-2 (1000 U/ml) and anti-CD16 mAbs (3 µg/ml) overnight before they were cultured with OCs and sAJ2 at a ratio of 1:2:4 (OCs:NK:sAJ2). Stain free Biorad technology was used to determine equal load of cell lysate on SDS-PAGE gel using IL-2 treated primary NK cells, and super-charged NK cells **(A)**. The supernatants were harvested from day 15 Snk cells and overnight IL-2 treated primary NK cells cultures and was used to determine IFN-γ secretion using single ELISA (n=10) **(B)**.

**Figure S3: Increased susceptibility of differentiated pancreatic tumor cell lines to chemotherapeutic drugs in comparison to their stem-like counterparts.**

MP2 cells were treated with IL-2 (1000 U/ml) and anti-CD16 mAbs (3µg/mL) treated NK cells supernatants to induce differentiation as described in Materials and Methods section. MP2 cells were also treated with anti-TNFα (1:100) and anti-IFNγ (1:100) for 18-20 hours. MP2, Diff-MP2, and anti-TNFα + anti-IFNγ treated MP2 cells were treated with cisplatin (60 µg/mL) for 18-20 hours, after which, the cells were stained with PI to determine percent cell death using flow cytometric analysis (n=3) **(A)**. MP2, PL12, and Capan were treated with cisplatin (60 µg/mL) for 18-20 hours, after which, the cells were stained with PI to determine percent cell death using flow cytometric analysis (n=3) **(B)**.

**Figure S4: Combination of sNK cell immunotherapy and CDDP increased IFN-γ secretion in spleen, peripheral blood, and bone marrow of hu-BLT mice.**

Hu-BLT mice were implanted with OSCSC tumors and, were injected with sNK cells and CDDP as shown in figures. At the end of experiment, hu-BLT mice were sacrificed spleens **(A)**, peripheral blood **(B)**, and bone marrow **(C)** were harvested; and single cell suspensions were obtained and cultured with IL-2 (1000 U/ml) for 7 days. On day 7, the supernatants were harvested and the secretions of IFN-γ were determined using single ELISA **(A, B, C)**.

**Figure S5: Combination of sNK cell immunotherapy and CDDP increased IFN-γ secretion and NK cell-mediated cytotoxicity in spleen, peripheral blood, and bone marrow of hu-BLT mice.**

Hu-BLT mice were implanted with OSCSC tumors and, were injected with sNK cells and CDDP as shown in figures. At the end of experiment, hu-BLT mice were sacrificed spleens, peripheral blood, and bone marrow were harvested; and single cell suspensions were obtained and cultured with IL-2 (1000 U/ml) for 7 days. On day 7, the supernatants were harvested and the secretions of IFN-γ were determined using single ELISA **(A)**. Spleens, peripheral blood, and bone marrow cells were cultured with IL-2 (1000 U/ml) for 7 days. On day 7, cells were used as effectors against OSCSCs using standard 4-hour ^51^Cr release assay. The Lytic units (LU) 30/10^6^ cells were determined using the inverse number of cells required to lyse 30% of OSCSCs x 100 **(B)**. One of three representative experiments is shown in figure.

**Figure S6: Differentiated susceptible of CSCs/poorly differentiated tumors to NK cell-mediated cytotoxicity when compared to their differentiated counterparts**

Purified NK cells (1×10^6^ cells/ml) from healthy individuals were left untreated, or treated with IL-2 (1000 U/ml), or treated with IL-2 (1000 U/ml) and anti-CD16 mAbs (3µg/mL) for 18 hours and were used as effectors in chromium release assay. OSCSCs were differentiated using IL-2 (1000 U/ml) and anti-CD16 mAbs (3µg/mL) treated NK cell supernatants as described in Materials and Methods section. OSCSCs, NK-differentiated-OSCSCs, and differentiated OSCCs were labeled with ^51^Cr for an hour after which cytotoxicity against the tumor cells was determined using a standard 4-hour ^51^Cr release assay. The Lytic units (LU) 30/10^6^ cells were determined using the inverse number of NK cells required to lyse 30% of tumors x 100 (n=3) **(A)**. Purified NK cells (1×10^6^ cells/ml) from healthy individuals were left untreated, or treated with IL-2 (1000 U/ml), or treated with IL-2 (1000 U/ml) and anti-CD16 mAbs (3µg/mL) for 18 hours and were used as effectors in chromium release assay. OSCSCs were differentiated using IL-2 (1000 U/ml) and anti-CD16 mAbs (3µg/mL) treated NK cell supernatants as described in Materials and Methods section. MP2, NK-differentiated-MP2, and differentiated PL12 were labeled with ^51^Cr for an hour after which cytotoxicity against the tumor cells was determined using a standard 4-hour ^51^Cr release assay. The Lytic units (LU) 30/10^6^ cells were determined using the inverse number of NK cells required to lyse 30% of tumors x 100 (n=3) **(A)**. ***(*p* value 0.0001-0.001), **(*p* value 0.001-0.01), *(*p* value 0.01-0.05).

**Figure S7: PD-L1 surface expression on NK cells, and MHC-class I surface expression on tumor cells.**

Purified NK cells (1×10^6^ cells/ml) from healthy individuals were left untreated, treated with IL-2 (1000 U/ml) or treated with IL-2 (1000 U/ml) and anti-CD16 mAbs (3µg/mL) for 18 hours before PD-L1 surface expression was determined using flow cytometer. IgG2 isotype antibodies were used as controls **(A)**. OSCSCs were differentiated using IL-2 (1000 U/ml) and anti-CD16 mAbs (3µg/mL) treated NK cells supernatants as described in Materials and Methods section. The surface expression level of MHC-class I was analyzed on OSCSCs, NK cell-differentiated OSCSCs, and OSCCs using flow cytometry. IgG2 isotype antibodies were used as controls **(B)**. MP2 cells were differentiated using IL-2 (1000 U/ml) and anti-CD16 mAbs (3µg/mL) treated NK cells supernatants as described in Materials and Methods section. The surface expression level of MHC-class I was analyzed on MP2, NK cell-differentiated MP2, and PL12 using flow cytometry. IgG2 isotype antibodies were used as controls **(C)**.

**Figure S8: Increased IFN-γ secretion by NK cells in the presence of cancer stem cells and anti-PD1.**

OSCSCs were differentiated using IL-2 (1000 U/ml) and anti-CD16 mAbs (3µg/mL) treated NK cells supernatants as described in Materials and Methods section. NK cells of healthy individual, pancreatic cancer patient (patient.1), and tonsillar cancer patient (patient.2) were treated with IL-2 (1000 U/ml) for 18-20 hours before NK cells and tumors cells were co-cultured (NK: tumors; 1:1), and were treated with anti-PD1 (500 ng/ml). On day 3 of co-culture, the supernatants were harvested and the secretions of IFN-γ were determined using single ELISA.

**Figure S9: Combination of super-charged NK cells with anti-PD1 antibody injection halted growth of MP2 tumors in hu-BLT mice.**

Successfully reconstituted hu-BLT mice were orthotopically injected with 1×10^6^ of human MP2 cells in the pancreas. One or two weeks after tumor implantation selected hu-BLT mice received 1×10^6^  super-charged NK cells via tail vein injection. Seven days later, anti-PD1 (50 µg/mice) was injected via tail vain injection. At the end of experiment, animals were sacrificed and pancreas pictures were taken post mortem **(A)**. The single cells suspensions were prepared from the mice’s pancreas as described in Materials and Methods, and were cultured for 7 days when the pictures of culture were taken **(B).**

**Figure S10: Combination of sNK cells and anti-PD1 antibody injection increased IFN-γ secretion and cytotoxic function by PBMCs, splenocytes and bone marrow derived immune cells in hu-BLT mice.**

Hu-BLT mice were orthotopically injected with 1 x 10^6^ human MP2 tumors in the pancreas. One week after the tumor implantation, mice received supercharged NK (sNK) cells via tail-vein injection, and one week after sNK cells injection, mice received anti-PD1 (50 µg/mice) via tail vein. The disease progression was monitored for another week. At the end of experiment, hu-BLT mice were sacrificed spleens, peripheral blood, and bone marrow were harvested; and single cell suspensions were obtained and cultured with IL-2 (1000 U/ml) for 7 days. On day 7, the supernatants were harvested and the secretions of IFN-γ were determined using single ELISA **(A)**. Spleens, peripheral blood, and bone marrow cells were cultured with IL-2 (1000 U/ml) for 7 days. On day 7, cells were used as effectors against OSCSCs using standard 4-hour ^51^Cr release assay. The Lytic units (LU) 30/10^6^ cells were determined using the inverse number of cells required to lyse 30% of OSCSCs x 100 **(B)**. One of three representative experiments is shown in figure.

**Figure S11: Combination of sNK cells and anti-PD1 antibody injection increased IFN-γ secretion and cytotoxic function by PBMCs, splenocytes and bone marrow derived immune cells in hu-BLT mice.**

Hu-BLT mice were orthotopically injected with 1 x 10^6^ human OSCSCs tumors in the floor of mouth. One week after the tumor implantation, mice received supercharged NK (sNK) cells via tail-vein injection, and one week after sNK cells injection, mice received anti-PD1 (50 µg/mice) via tail vein. The disease progression was monitored for another week. At the end of experiment, hu-BLT mice were sacrificed spleens, peripheral blood, and bone marrow were harvested; and single cell suspensions were obtained and cultured with IL-2 (1000 U/ml) for 7 days. On day 7, the supernatants were harvested and the secretions of IFN-γ were determined using single ELISA, and cells were used as effectors against OSCSCs using standard 4-hour ^51^Cr release assay. The Lytic units (LU) 30/10^6^ cells were determined using the inverse number of cells required to lyse 30% of OSCSCs x 100.

**Table S1**

|  | **Anti-CD16** | **Anti-PD1** | **Anti-PDL1** |
| --- | --- | --- | --- |
| **Origin** | Mouse | Mouse | Mouse |
| **Mode of Action** | Activation | Activation | Activation |
| **Catalog number** | 302001 | 14-9985-82 | 16-5983-82 |
| **Vendor** | Biolegend (San Diego, CA, USA) | Invitrogen (Carlsbad, CA, USA) | Invitrogen, (Carlsbad, CA, USA) |

**Figure S1**

**A**

**B**

**C**

**D**

**
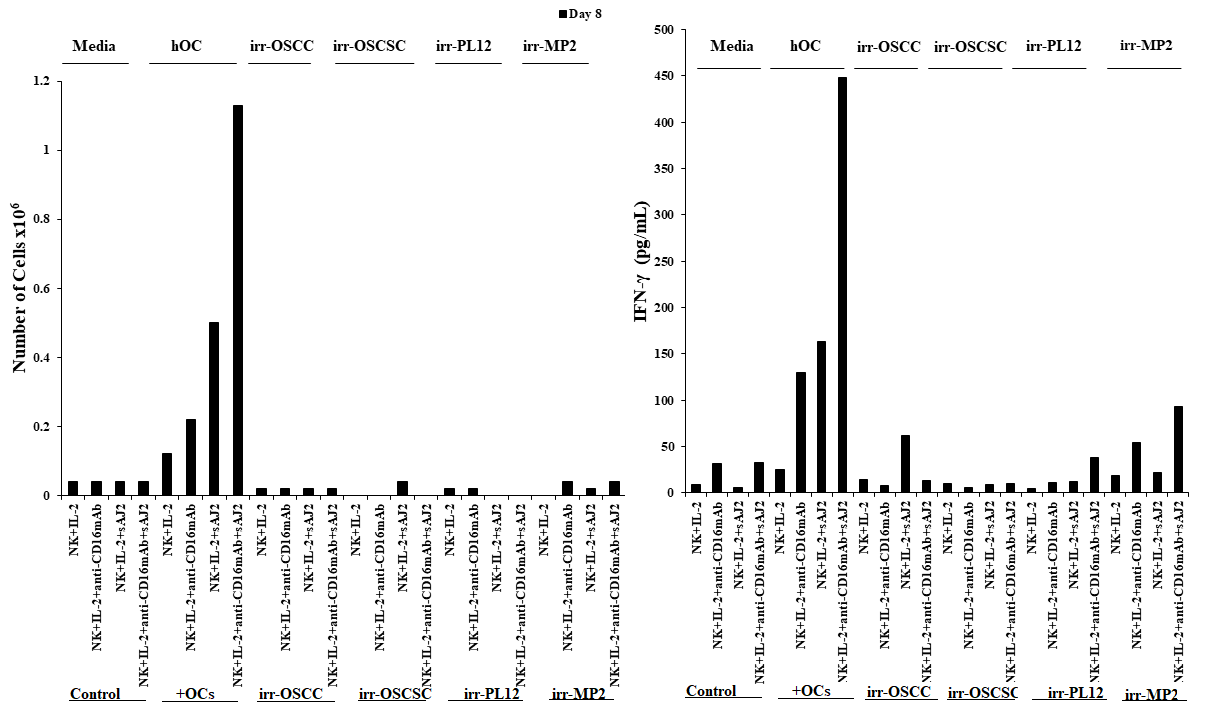
**

**E**

**Figure S2**

**A B**


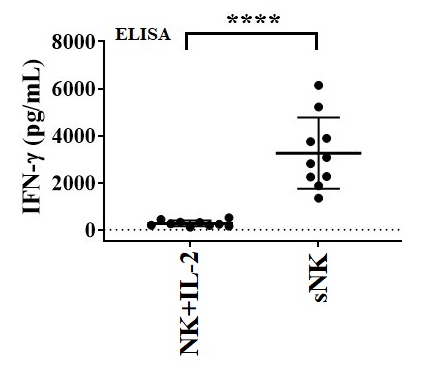

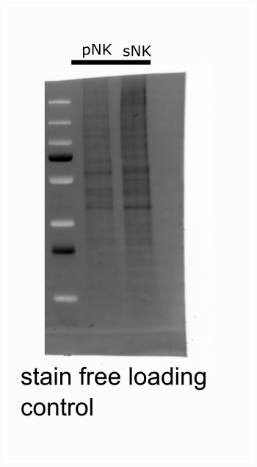


**Figure S3**

**Figure S4**


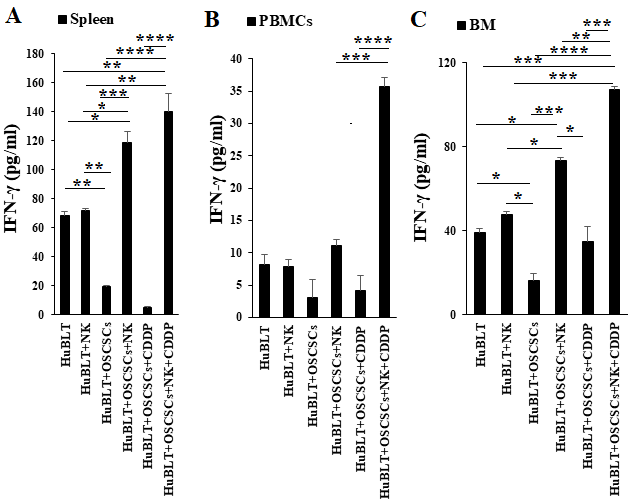


**Figure S5**

**
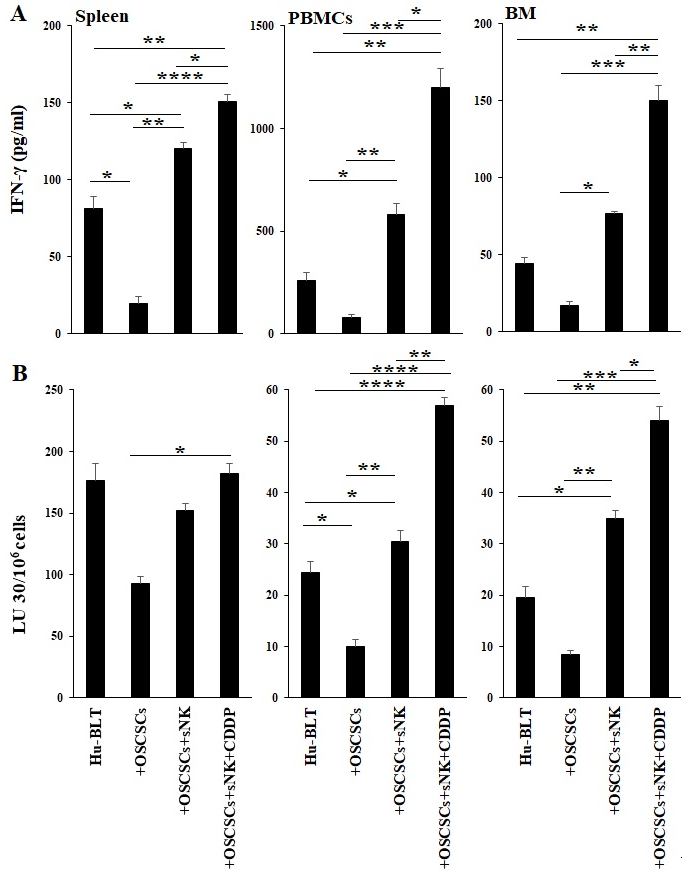
**

**Figure S6**

**A**

**B**

**Figure S7**

**A**

**
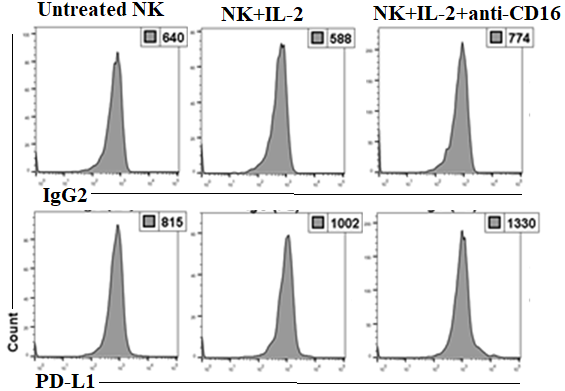
**

**B**

**C**

**Figure S8**

**Figure S9**


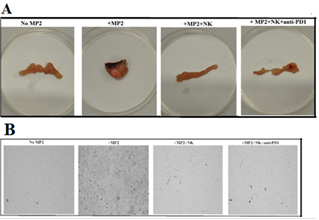


**Figure S10**

**
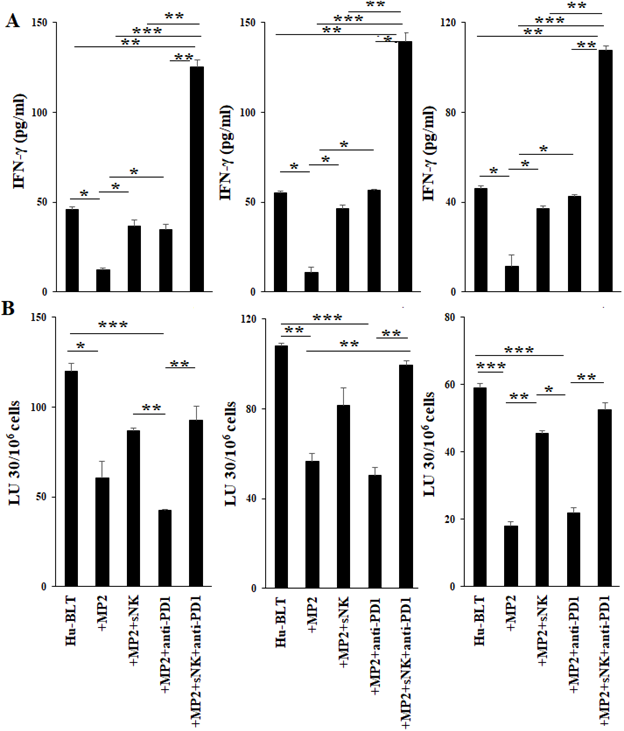
**

**Figure S11**

**
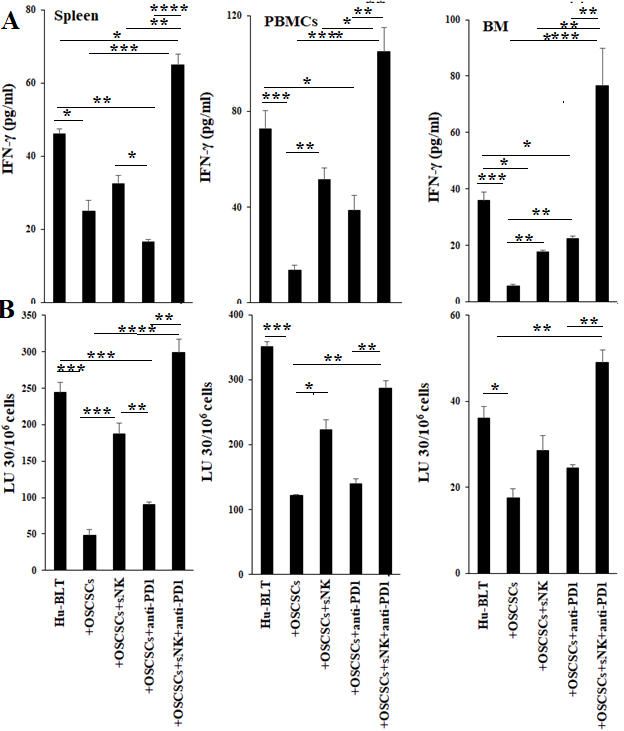
**
